# Thermoplasmonic Nano–Hybrid Core@Shell Ag@SiO_2_ Films Engineered via One–Step Flame Spray Pyrolysis

**DOI:** 10.3390/nano15100743

**Published:** 2025-05-15

**Authors:** Christos Dimitriou, Yiannis Deligiannakis

**Affiliations:** Laboratory of Physical Chemistry of Materials & Environment, Department of Physics, University of Ioannina, 45110 Ioannina, Greece; ch.dimitriou@uoi.gr

**Keywords:** thermoplasmonic, heat dissipation, flame spray pyrolysis, impinging, Ag@SiO_2_, in situ film deposition, photothermal efficiency, fine-tuning thickness

## Abstract

Thermoplasmonic heat generation by silver (Ag) nanoparticles can harness visible light to efficiently produce localized heating. Flame spray pyrolysis (FSP) is a powerful one-step synthesis technology for fabricating plasmonic Ag-based nanostructures. In the present study, we employed FSP to engineer core@shell Ag@SiO_2_ nanoparticles coated with an ultrathin (1–2 nm) silica (SiO_2_) nanolayer in a single step *in tandem* with their deposition as films onto solid substrates. Accordingly, we engineered a library of Ag@SiO_2_ nanofilms with precisely controlled thicknesses in the range of 1–23 μm. A systematic study of the thermoplasmonic heat-generation efficiency (ΔT) of the films under visible-light irradiation (LED, λ = 405 nm) revealed that the films’ compactness and thickness are key parameters governing the heat-generation efficiency and thermal response rate. Moreover, we show that the substrate type can also play a key role; Ag@SiO_2_ films on glass-fiber filters (PGFFs) enabled faster temperature increase (dT/dt) and a higher maximum temperature gain (ΔT_max_) compared with Ag@SiO_2_ films on glass substrates (PGSs). The photothermal conversion efficiencies were approximately 60%, with the highest efficiency (η = 65%) observed in the thinner impinged film. This study demonstrates that FSP-derived Ag@SiO_2_ nanofilms provide a versatile and scalable platform for thermoplasmonic heat generation applications with significant industrial potential.

## 1. Introduction

Nanoplasmonics are based on nanomaterials able to interact with electromagnetic waves *via* collective oscillations of their free electrons, termed surface plasmons, at metal–dielectric interfaces [1]. At the nanoscale, localized surface plasmon resonance (LSPR) [2,3] can be dominant, characterized by either (i) radiative or (ii) nonradiative processes: In the former, radiative decay involves light scattering by plasmonic particles after photoexcitation, producing the vivid colors seen in noble metals [4]. This process can include the photoexcitation/transfer of high-energy *hot electrons* to suitable acceptors [5,6,7,8] and the formation of localized enhanced electric near-fields, or so-called *hot spots* [9,10], due to the confinement of surface plasmons and incident photons near the particle. In nonradiative processes, nonradiative decay entails the conversion of electromagnetic energy into thermal energy, leading to localized heating. This effect has given rise to *thermoplasmonics* [11], a subfield that leverages the photothermal properties of metallic nanoparticles for various applications.

Plasmonic materials, such as silver (Ag^0^), gold (Au^0^), and copper (Cu^0^) exhibit unique optical properties due to their ability to support surface plasmon resonances [1,12]. Among these, Ag^0^ is often regarded as the most efficient plasmonic material, particularly in the visible and near-infrared regions [13,14,15]. Additionally, Ag^0^ nanoparticles demonstrate higher refractive-index sensitivity, making them highly responsive to changes in the surrounding environment—an advantageous property for applications such as sensing. However, a key drawback is that Ag^0^ is susceptible to oxidation and tarnishing, which can degrade its plasmonic performance over time. To mitigate this, silver nanoparticles are often encapsulated with silica (SiO_2_) shells, resulting in core@shell Ag@SiO_2_ nanoplasmonic hybrids. This silica coating acts as a protective barrier, preventing oxidation and preserving the nanoparticles’ optical properties [8,16,17,18]. Moreover, the SiO_2_ shell offers a versatile platform for further functionalization [8,16,19,20] and enables precise regulation of the spatial arrangement of adjacent Ag^0^ particles, thereby allowing fine-tuning of the interparticle distance, which is crucial for plasmonic and photonic applications.

So far, thermoplasmonics have found applications across multiple disciplines [21]. In biomedicine, these methods enable targeted photothermal therapy for cancer treatment and facilitate drug delivery systems [18,22,23,24,25,26]. Recently, due to the COVID-19 pandemic, thermoplasmonic-assisted plasmonic biosensors have been developed for sensitive and rapid detection of SARS-CoV-2 [27,28]. In chemistry, thermoplasmonics can enhance catalytic reactions through localized heating [29,30,31,32,33]. Furthermore, they can enhance solar energy harvesting by improving the efficiency of photovoltaic devices [34,35,36].

Plasmonic materials can be synthesized using a wide variety of methods, including sol–gel [37], hydrothermal synthesis [38], electrochemical deposition [39], vapor-phase deposition [40], and template-guided self-assembly [41]. Here, we used flame spray pyrolysis (FSP) technology [42,43,44] to engineer Ag@SiO_2_ nanoplasmonics and deposit them as micrometric films on solid substrates. The key aim was to develop Ag@SiO_2_ films via one-step FSP, optimized for thermoplasmonic heat generation. In our recent review [42], we reported various FSP configurations and experimental setups that enable the synthesis of diverse multifunctional nanomaterials and nanodevices, including thin films. However, very few studies have thus far focused on plasmonic films engineered *in situ* using FSP technology. Li et al. developed FSP-made SERS-sensing substrates by depositing plasmonic nanoaggregates of Ag and SiO_2_ on glass substrates, enabling rapid and quantitative detection of pesticide residues in food [45,46]. Bletsa and co-workers produced durable, visible-light-active photocatalytic Ag/TiO_x_–polymer nanocomposite films for medical devices, enabling repeated on-demand biofilm eradication without intrinsic cytotoxicity [47]. Blattmann et al. presented an FSP-based scalable approach for *in situ* synthesis of Ag-polymer films, enabling the optimization of conductive network formation for flexible electronic applications [48,49]. Merkl et al. [24] investigated single-step aerosol self-assembly of plasmonic Ag-SiO_2_ nanofilms with tunable extinction from the visible to near-infrared (NIR) region. This tunability was achieved by incorporating silica (SiO_2_) during flame synthesis—not as a coating, but as a co-agglomerated dielectric spacer. The photothermal properties of these NIR-responsive (λ = 808 nm) nanocomposite films were exploited to eradicate established biofilms of clinically relevant *Escherichia coli* and *Staphylococcus aureus* [24]. Nonetheless, despite these few studies, the engineering of FSP-made plasmonic films tailored for thermoplasmonic applications remains underexplored. In our previous work, we demonstrated that FSP-made Ag@SiO_2_ nanoparticles in powder form and subsequently drop-cast onto glass substrates exhibited superior thermoplasmonic heat-generation efficiency [50].

The specific aims of this work were [a] to use flame spray pyrolysis (FSP) technology [42,43,44] to synthesize silver (Ag) nanoparticles encapsulated by an ultrathin (1–2 nm) silica (SiO_2_) shell in a single step; [b] to employ FSP for *in situ* deposition of these Ag@SiO_2_ structures onto solid substrates—namely, either glass substrates or glass fiber filters—forming films with controlled thicknesses ranging from 1 to 23 μm; and [c] to study the thermoplasmonic heat generation efficiency (ΔT) of these films under visible-light irradiation (λ = 405 nm). Our results highlight the critical influence of film compactness on heat generation efficiency and thermal response. Thus, the present work establishes FSP-made Ag@SiO_2_ nanofilms as a scalable and adaptable platform for thermoplasmonic heat generation, highlighting their suitability for industrial applications.

## 2. Materials and Methods

### 2.1. In-Situ Film Deposition Engineering via Flame Spray Pyrolysis

**Figure 1:**
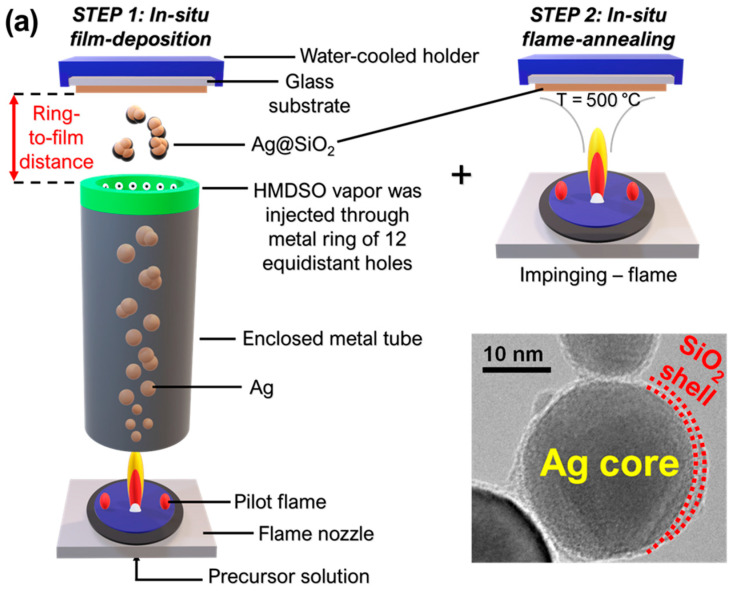

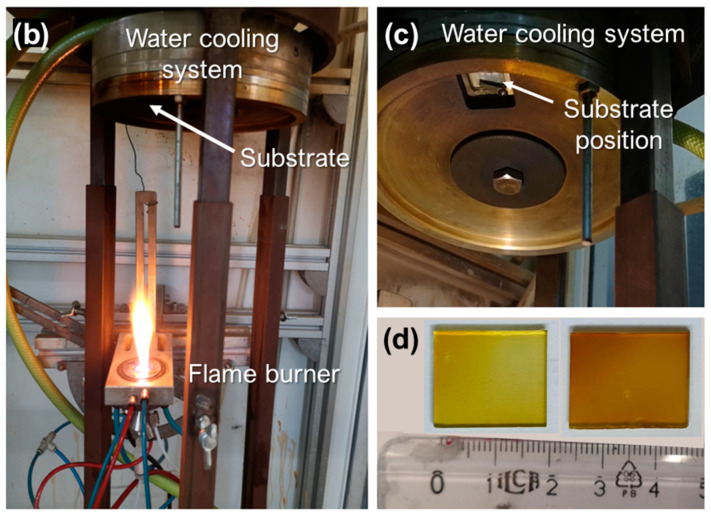
(**a**) Schematic representation of in situ film deposition via flame spray pyrolysis (FSP), illustrating (Step 1) nanoplasmonic Ag@SiO_2_ film deposition onto a glass substrate and (Step 2) impinging flame annealing of the deposited film. *Inset*: TEM image of Ag@SiO_2_ powder showing a SiO_2_ shell thickness of 1–2 nm. (**b**) Photograph of the FSP film deposition setup. (**c**) Water-cooled holder during FSP process, also showing the substrate position above the flame. (**d**) Demonstration of successful Ag@SiO_2_ film deposition.

A flame spray reactor was used to produce nanoplasmonic silver silica-coated Ag@SiO_2_ (core@shell) particles by the flame spray pyrolysis (FSP) method [50,51,52]. The precursor solution was prepared by dissolving silver acetate (Sigma-Aldrich, St. Louis, MO, USA, purity > 99%) in a mixture of 2-ethylhexanoic acid (EHA) and acetonitrile (ACN) (both Sigma-Aldrich, purity > 97%) at a 1:1 volume ratio to achieve a concentration of 0.4 M. The solution was delivered through a capillary at a rate of 5 mL/L and dispersed with oxygen (O_2_, Linde, Dublin, Ireland, purity > 99%) at 5 L/min into a stoichiometric, self-sustained oxygen–methane pilot flame (O_2_: 5 L/min, CH_4_: 2.5 L/min) to initiate combustion, resulting in the formation of metallic silver nanoparticles (Ag^0^). A pressure drop of 2 bar was maintained at the nozzle tip, while an additional 10 L/min of sheath O_2_ was introduced to stabilize the flame. *In situ* silica (SiO_2_) coating of Ag nanoparticles was performed in a modified enclosed FSP reactor, as originally described by Sotiriou and co-workers [16,18,51,53]. The FSP flame was confined within a 22 cm-long metallic tube (Figure 1a) hermetically sealed at the burner and capped by a metal ring (diameter = 4.3 cm) that featured 12 equidistant holes (0.5 cm in diameter) directed away from the centerline and oriented downstream to prevent stagnation flow.

Hexamethyldisiloxane (HMDSO, Aldrich, purity 98%) vapor was introduced into the flame *via* 0.5 L/min nitrogen (N_2_, Linde, purity > 99%), supplemented by an additional 15 L/min of N_2_ at room temperature, introducing the necessary swirl to enhance the coating efficiency. The HMDSO vapor was generated by bubbling N_2_ through 300 cm^3^ of HMDSO in a glass flask maintained at 5 °C. According to the SiO_2_ coating methodology via FSP, using the Antoine equation for HMDSO vapor pressure [54], our setup yielded a theoretical SiO_2_ production rate of 1.78 g/h, corresponding to 6 wt% SiO_2_ in the final product powder. As reported by Moularas et al. [50] and Teleki et al. [52,55], for this specific SiO_2_ content in the matrix, the SiO_2_ thickness was 1–2 nm (see TEM image in Figure 1a). This is further corroborated by the EDS spectrum of a representative PGS sample in the Supporting Information, showing the presence of both Ag and Si (Figure S1). We deliberately targeted this SiO_2_ thickness to position the silver (Ag^0^) nanoparticles in close proximity, thereby maximizing the neighboring Ag^0^–Ag^0^ thermoplasmonic efficiency. This arrangement enhanced the collective photothermal effect, as closely spaced Ag nanoparticles can exhibit strong plasmonic coupling, leading to increased heat generation upon light irradiation [50].

The so-produced Ag@SiO_2_ particles were directly deposited as a film (see Figure 1) onto either [i] a glass substrate (commercial soda–lime glass) or [ii] a glass fiber filter (Albet Labscience GF6 257 mm in diameter, Hahnemühle FineArt GmbH, Dassel, Germany) [56] using a vacuum pump (Busch V40, Virginia Beach, VA, USA), corresponding to the conventional FSP setup typically used for collecting FSP-made particles. After deposition, the glass fiber filter with Ag@SiO_2_ nanoparticles adhered to its fibers was processed by scraping off excess material to obtain the desired sample, hereafter referred to as plasmonic glass fiber filters (PGFFs).

A schematic of the FSP apparatus for *in situ* film deposition onto glass substrates is detailed in Figure 1. The configuration used was identical to a standard single-nozzle FSP setup, with the addition of a water-cooled glass substrate holder (see Step 1 in Figure 1a). Thermophoresis was employed to achieve the direct deposition of freshly formed nanoparticles onto the glass substrate. In the context of nanoparticle deposition, thermophoresis facilitates the directed motion of nanoparticles toward a cooler substrate. The temperature difference between the flame and the substrate creates a thermal gradient, causing the hotter nanoparticles within the flame to migrate upwards, toward the cooler surface due to thermophoretic forces. This selective migration enables the direct deposition of nanoparticles onto the substrate, leading to the formation of thin films or coatings.

To control nanoparticle sintering, the optimal height above the burner (HAB) was selected. Films produced at HAB ≥ 10 cm exhibited low density and poor mechanical adhesion [57]. Therefore, to overcome this limitation, a post-deposition thermal-annealing step, “impinging”, was applied (see Step 2 in Figure 1a). This approach was based on the use of an FSP flame operated without a metal precursor—i.e., solvent-only—to “impinge” the nanoparticle film. As demonstrated *via* SEM imaging hereafter, this method proved to be highly effecient, enabling a simple, one-step optimization of the film’s compactness. This technique of FSP-impinging was previously used by Kühne et al. and Tricoli et al. for FSP-micromachined single-chip gas sensors [58,59]. Initially, the deposition followed an in-flame agglomeration regime due to the low substrate temperature. Duringimpinging, sintering occurred [60], leading to the transformation of porous structures into compact film morphologies and a significant decrease in the specific surface area (SSA). Thus, FSP deposition/annealing allowed the fabrication of stable, micro-patterned functional inorganic nanomaterial layers with enhanced durability [61].

Hereafter, the SiO_2_-spraying ring-to-substrate distance was fixed at 5 cm, resulting in a total HAB (height above burner) of 27 cm. Six different deposition times were investigated: t_deposition_ = 15, 45, 60, 90, 180, and 360 s, resulting in films of varying thicknesses. These materials were coded as plasmonic glass substrates (PGSs), listed in Table 1. Subsequently, for the *in situ* flame annealing process, the “impinging” (see Step 2 in Figure 1a), an ethanol-only spray flame was utilized for 30 s, with a solvent-feed rate of 11 mL/min, dispersed with oxygen at 3 L/min into a pilot flame (O_2_: 4 L/min, CH_4_: 2 L/min). For the PGFFs, two different impinging times were used: t_impinging_ = 60 and 120 s listed in Table 1. The films were positioned at a HAB = 20 cm, ensuring the film stood just a few centimeters above the edge of the flame to prevent damage from the flame flow. The surface temperature of the film was measured to be T_PGS_ = 500 °C, while that of the fiber filter was T_PGFF_ = 350 °C.

### 2.2. Characterization of the Ag@SiO_2_ Nanofilms

*X-ray Diffraction*: X-ray diffraction (XRD) data were acquired at room temperature using a Bruker D8 Advance 2theta diffractometer (Bruker AXS GmbH, Karlsruhe, Germany) equipped with copper radiation (Cu Kα, *λ* = 1.5406 Å) and a secondary monochromator (operating at 36 kV, 36 mA). Measurements were conducted over the 2θ range from 10° to 80°. Crystal size was determined using the Scherrer formula (Equation (1)) as follows [62]:

(1) $$d_{\mathrm{XRD}}=\frac{K\;\lambda }{\mathrm{FWHM}\;\times \cos\theta }$$

where *K* = 0.9, and FWHM represents the full width at half-maximum of the XRD peaks. The XRD patterns were recorded in standard Bragg–Brentano geometry using a step-scanning mode with a step size of 0.003° and a counting time of 1 s per step.

*Ultraviolet-Visible (UV-Vis) Spectroscopy*: UV-Vis spectra of the nanoplasmonic films were acquired using a PerkinElmer Lambda 35 spectrometer (PerkinElmer, Inc., Shelton, CT, USA), operating over a wavelength range of 200–800 nm with a spectral bandwidth of 2 nm. During measurements, all films were positioned vertically in the optical path to ensure precise interaction with the incident light. The instrument featured a holographic concave grating with 1053 lines/mm as the monochromator, while pre-aligned deuterium and tungsten–halogen lamps provided continuous spectral coverage across the UV and visible regions.

*Scanning Electron Microscopy (SEM)*: The morphology, microstructural characteristics, and thickness of the nanoplasmonic Ag@SiO_2_ films deposited either on glass substrates or glass fiber filters were analyzed using a JEOL JSM-IT210 scanning electron microscope (JEOL Ltd., Akishima, Tokyo, Japan). The samples were mounted on aluminum stubs using conductive carbon tape and examined under high-vacuum conditions ( ~ 10^−4^ Pa) without any additional coating. Imaging was performed at an accelerating voltage of 10–25 kV with a working distance ~ 11 mm, utilizing secondary electron (SE) and backscattered electron (BSE) detectors to capture topographical and compositional contrast, respectively. Film thickness was determined from cross-sectional SEM images (Figure 3) obtained by cleaving the samples and imaging the exposed edge. For each film, three independent cross-sections were analyzed to ensure completeness and statistical relevance.

### 2.3. Monitoring Plasmonic Heating (ΔT) Dynamics

Temperature measurements were recorded using an infrared thermal imager (Fluke TiS40, Fluke Corporation, Everett, WA, USA). The emissivity was set to 0.95 and the background temperature was maintained at 23 °C (room temperature). Emissivity differences between substrates were considered and corrected during the analysis to ensure accurate temperature comparisons. Data analysis was performed using SmartView software. As a control, the maximum temperature increase in glass substrates without Ag@SiO_2_ nanoparticles was ΔT_max_ = 13 ± 1 °C, while in blank glass fiber filters, it was ΔT_max_ = 8 ± 1 °C (see thermal images of blank glass substrate and a blank glass fiber filter in Figure S2, Supporting Information). The standard error values shown in the temperature curves correspond to the thermal camera’s accuracy range (±1 °C).

*Substrate Irradiation*: The radiative source used was a FireJet FJ100 72 × 20 AC LED lamp from Phoseon Technology Inc. (NE Evergreen Parkway, Hillsboro, OR, USA), with a specific wavelength of 405 ± 10 nm. The nanoplasmonic films and glass fibers were irradiated within an improvised enclosed box (Figure S3). The samples were placed on a resin base to ensure they faced the LED illumination vertically and were fixed at the focal point of the beam. The beam entered the irradiation compartment through a guiding tube positioned at the end of the LED. The incident irradiation power at the sample position was calibrated using a power meter (Newport model 1918-c) and was fixed at 0.14 ± 0.01 W/cm^2^. The lamp’s power profile is presented (Figure S4). All irradiation experiments were repeated three times to verify the precision of the collected data.

### 2.4. Theoretical Background

To further refine our understanding, we discuss theoretical aspects of the temperature increase (ΔT) in a typical 2D array geometry. The pioneering work of Baffou et al. [13,63] in the field of thermoplasmonics provides a theoretical framework for interpreting the temperature increase (ΔT) in an ensemble of N nanoparticles arranged in a regular array with an interparticle distance p. This temperature increase arises from two distinct contributions: (i) *self-contribution*, attributed to the heat generated by each nanoparticle individually, and (ii) *collective contribution* resulting from thermal interactions with neighboring nanoparticles. Under uniform irradiation, the total rise in temperature (ΔT) for such an ensemble of nanoparticles deposited on a planar substrate—specifically, in our case, a 2D finite-size square film—can be described by the following closed-form expression (2):

(2) $$\Delta T\;(r,\;p)={\Delta T}_{\mathrm{self}\;}\left(r\right)+{\Delta T}_{\mathrm{coll}\;}\left(p\right)=\frac{\sigma_{\mathrm{abs}\;}I}{4\pi \kappa r}+\frac{\sigma_{\mathrm{abs}\;}I}{\kappa }\;\frac{\ln(1+\sqrt{2})}{\pi }\;\frac{S}{p^{2}}$$

where *σ*_abs_ is the absorption cross-section of the nanoparticle (m^2^), *I* (*λ*) the incident light intensity (W/m^2^) at a particular wavelength (*λ*), *κ* the thermal conductivity of the embedding medium (W m^−1^ K^−1^), *r* the distance from the heat source (m), *S* the total area of the 2D film (m^2^), and *p* the interparticle distance (m). It is important to note that the product *σ*_abs_ · *I* corresponds to *Q*, the heat power (W) generated by a single nanoparticle [13,63].

To determine the time-dependent temperature profile of the 2D finite-size film under uniform irradiation, we focus on the collective contribution, as the densely packed nanoparticles strongly influence each other’s heat generation [13,63]. This interaction leads to a continuous and dominant temperature field across the film, making the self-contribution negligible. To describe this thermal evolution, we consider a point source in a surrounding medium with thermal diffusivity α_eff_, delivering constant heat power. The temperature evolution can be formulated using Green’s function formalism [13,63], which, through mathematical transformation, can be recast into a closed-form expression (3) involving the error function [13,63], as follows:

(3) $${\Delta T}_{\mathrm{heating}}\;(p,\;r,\;t)=\frac{\sigma_{\mathrm{abs}\;}I}{\kappa_{\mathrm{eff}}}\;\frac{\ln(1+\sqrt{2})}{\pi }\;\frac{S}{p^{2}}\;\left\{1-\mathrm{erf}\left(\frac{r}{2\sqrt{\alpha_{\mathrm{eff}\;}t}}\right)\right\}\;$$

where α_eff_ is the effective medium’s thermal diffusivity, α_eff_ = *κ*_eff_/(ρ_eff_ · c_p,eff_) (m^2^/s), ρ_eff_ is the effective density, and c_p_ is the effective specific heat capacity [13,63]. Each effective property is determined by averaging the corresponding properties of the constituent materials. For instance, in the case of PGSs, there is a glass substrate with air above it, while in PGFFs, there are glass fibers surrounded by air. According to Equation (3), the distance from the heat source (r) and the thermal properties of the surrounding medium govern the macroscopically observed ΔT kinetics. 

Now, let us consider the opposite scenario, where the illumination ceases, leading to the cooling phase of the system. In this case, the thermal energy dissipates into the surrounding medium, and the temperature profile follows a reverse evolution governed by heat diffusion. This cooling process can be described by the same heat transfer formalism but with a decaying temperature field, which is characterized by the system’s thermal diffusivity and boundary conditions [13,63]. The transient temperature evolution during cooling is given as follows:

(4) $${\Delta T}_{\mathrm{cooling}}\;(p,\;r,\;t)=\frac{\sigma_{\mathrm{abs}\;}I}{\kappa_{\mathrm{eff}}}\;\frac{\ln(1+\sqrt{2})}{\pi }\;\frac{S}{p^{2}}\;\mathrm{erf}\left(\frac{r}{2\sqrt{\alpha_{\mathrm{eff}\;}t}}\right)\;$$

## 3. Results and Discussion

### 3.1. FSP Engineering of Ag@SiO_2_ Films on Plasmonic Glass Substrates (PGSs) and Plasmonic Glass Fiber Filters (PGFFs)

**Figure 2:**
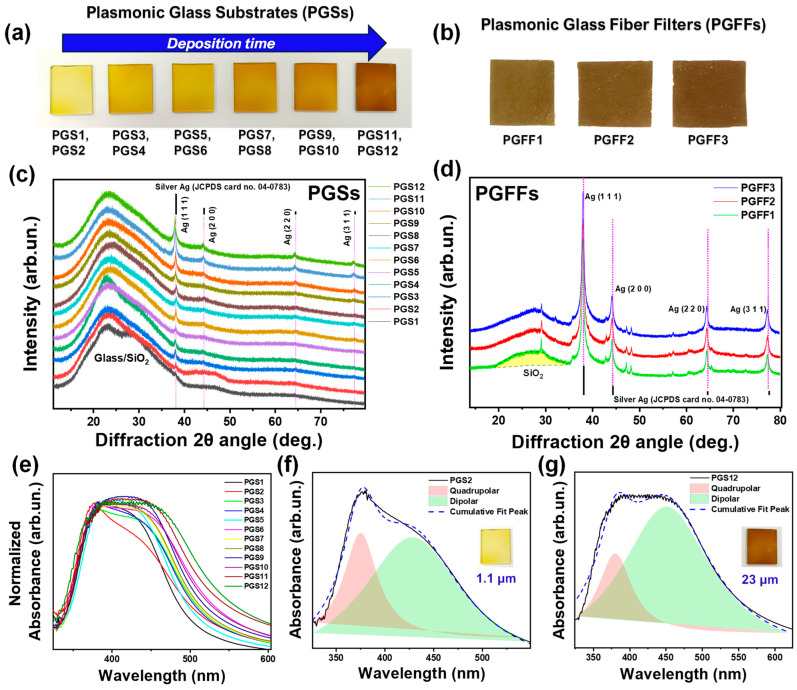
(**a**,**b**) Actual photographs of (**a**) Ag@SiO_2_ plasmonic glass substrates (PGSs), where each PGS image represents both non-impinged and impinged material as the color remains unchanged, and (**b**) Ag@SiO_2_ plasmonic glass fiber filters (PGFFs). For more information, see Table 1. (**c**,**d**) XRD patterns of (**c**) the twelve PGSs (non-impinged and impinged for six different deposition times) and (**d**) the three PGFFs (non-impinged and impinged for two different time periods). (**e**–**g**) UV-Vis spectra of Ag@SiO_2_ PGSs. (**e**) Normalized absorbance spectra of all PGSs. (**b**,**c**) Deconvoluted spectra of (**f**) PGS2 and (**g**) PGS12 impinged materials, illustrating the quadrupolar and dipolar contributions to the plasmonic bands.

In Figure 2a,b, we present actual photographs of the FSP-derived Ag@SiO_2_ PGSs and Ag@SiO_2_ PGFFs with increasing deposition time, t_deposition_. As shown in Figure 2a, we observed that with increasing t_deposition_, the films became progressively darker due to increased thickness. For further details on this, refer to the SEM results presented later. Correspondingly, Figure 2c,d display the XRD data of PGSs and PGFFs, showing the formation of metallic silver (Ag^0^) nanoparticles in all cases. The diffraction peaks can be indexed to the face-centered cubic (fcc) phase of silver (Ag^0^), with lattice parameters *a* = *b* = *c* = 4.086 Å (JCPDS card no. 04–0783) [64]. These peaks correspond to the (111), (200), (220), and (311) crystal planes of Ag^0^, with crystallite sizes ranging from 20 to 26 nm, as listed in Table 1. Furthermore, the typical amorphous SiO_2_ hump (from the glass and/or the SiO_2_ nano-shell) is resolved in the PGSs. In the PGFFs, minor quantities of elements typically found in borosilicate glass fibers (Figure 2d), such as boron (B), sodium (Na), and aluminum (Al) [56] were detected (verified by a control XRD on a non-treated glass fiber filter). As observed for the PGSs, increasing the deposition time on the glass substrate resulted in a crystallinity that remained almost constant, with small variations in the crystallite size (20–26 nm), while for the PGFFs, the crystallite size remained consistent in the range of 20–21 nm.

Impinging (see Step 2 in Figure 1a) promotes additional coalescence of particles on the preformed film, which can lead to an increase in particle size, depending on the substrate temperature. Specifically, such flame-annealing events (T_PGS_ = 500 °C for t_impinging_ = 30 s and T_PGFF_ = 350 °C for t_impinging_ = 60, 120 s) promoted a modest size increase of Ag^0^ particles by 1–4 nm (see Table 1).

**Figure 3:**
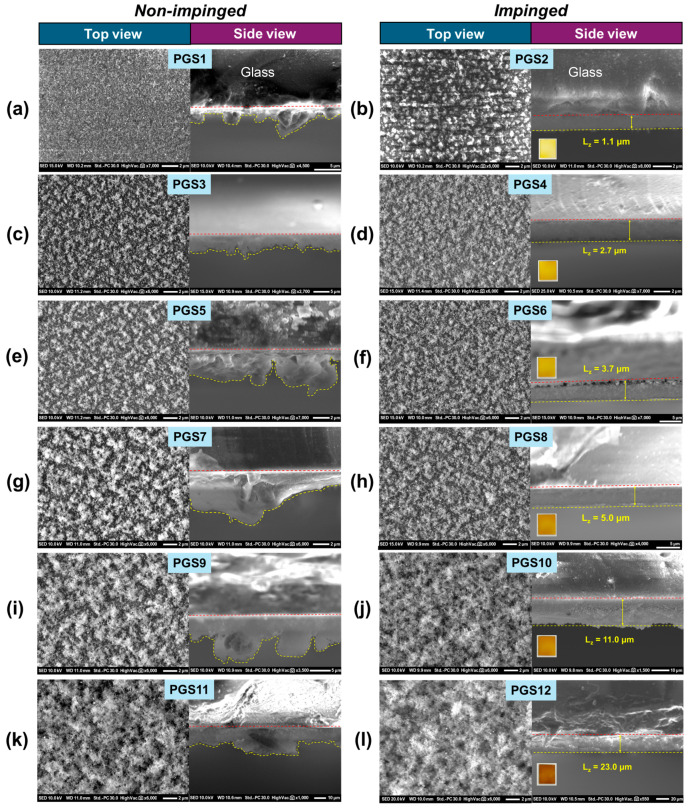
SEM images of Ag@SiO_2_ PGSs. Specifically, panels (**a**–**l**) show the PGS1–PGS12 materials, with each PGS presented in two views: the top view (**left**) and the side view of the glass substrate (**right**) providing information on film thickness.

Figure 2e presents the normalized UV-Vis spectra of all the PGS materials. The localized surface plasmon resonance (LSPR) for isolated Ag^0^ nanoparticles typically occurs at approximately 390 nm [65]. In our PGS samples, the resonance peak appeared between 385 and 390 nm. However, a noticeable broadening of the LSPR peak toward longer wavelengths was observed. PGSs with longer deposition times (resulting in greater thicknesses, see Figure 3) exhibited a pronounced red-shifted LSPR peak, attributable to Ag^0^–Ag^0^ coupling. As we recently analyzed in detail [50] in ensembles of non-monodisperse Ag^0^ nanoparticles, multiple LSPR peaks and band broadening reflect the extent of nanoparticle aggregation [4,66].

According to Mie theory [67], our Ag@SiO_2_ nanohybrids featuring 20–26 nm Ag cores encased in a 1–2 nm SiO_2_ shell can support both dipolar (D_0_) and quadrupolar (Q_0_) plasmonic modes [68,69,70], each influencing the thermoplasmonic behavior in distinct way: Figure 2f,g illustrate the deconvoluted UV-Vis spectra for two impinged films; the sparsely packed PGS2 (1.1 μm thickness) and the densely packed PGS12 (23 μm thickness), where both dipolar and quadrupolar plasmonic modes were observed. Dipolar plasmonic (“bright”) modes correspond to the collective oscillation of conduction electrons *in resonance* with incident light, leading to strong coupling with the electromagnetic field and efficient far-field scattering. These modes predominantly facilitate light absorption, generating hot electrons that drive photothermal heating [68,69,71]. In contrast, quadrupolar plasmonic (“dark”) modes exhibit more complex oscillation patterns with reduced far-field coupling, resulting in weaker scattering and absorption. Nevertheless, in densely packed nanoparticle assemblies—such as our Ag@SiO_2_ nanoparticles with a thin silica shell—near-field interactions can enhance quadrupolar-mode excitation [68,69,71]. While these modes contribute to localized heating, their overall influence on thermoplasmonic performance is typically expected to be less pronounced than that of dipolar modes [72]. As a result, in denser films (Figure 2g), the dipolar mode becomes more prominent than the quadrupolar mode. The excitation of dipolar and quadrupolar modes is influenced by nanoparticle size and interparticle spacing. In our Ag@SiO_2_ films, the thin silica shell allows strong near-field coupling between adjacent Ag cores, facilitating the excitation of both dipolar and quadrupolar modes. This near-field coupling can lead to hybridized plasmonic modes, altering the optical and thermal properties of the nanoparticles.

Figure 3 displays SEM images for all PGS films, with each PGS shown from two perspectives: the top view (left) and the side view of the glass substrate (right), which provide information on the Ag@SiO_2_ film thickness. For completeness, SEM images of a blank glass substrate and a blank glass fiber filter are presented for comparison (see Figures S3 and S4, respectively). To understand the role of the impinging process, Figure 3a,c,e,g,i,k present the non-impinged PGS films, where irregularities and inhomogeneity in the side-view film thicknesses can be clearly observed. The top views of the surfaces reveal larger Ag aggregates as the deposition time increases. In contrast, Figure 3b,d,f,h,j,l show the impinged PGS films; as the deposition time increases from 15 to 360 s, the surface becomes increasingly populated with Ag@SiO_2_ nanoparticles, leading to a more aggregated surface.

**Figure 4:**
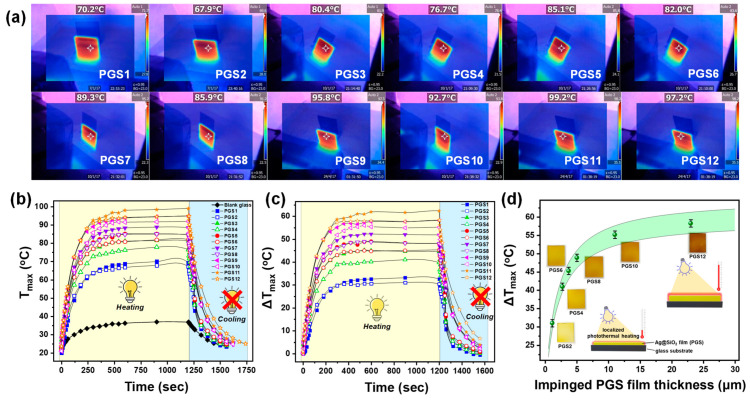
(**a**) Thermal images of all PGS materials, showing the localized maximum temperature (T_max_). (**b**) Kinetics plot of maximum temperature (T_max_) versus time for both heating (light ON from 0 to 1200 s) and cooling (light OFF from 1200 to 1600 s) processes for all PGSs. (**c**) Time–temperature profile (normalized to the blank glass substrate) for all PGS materials. (**d**) Maximum ΔT profile for various PGS film thicknesses, considering only impinged films with homogeneous surfaces. The green-shaded band represents the empirical–theoretical approach given by Equation (5), with data showing agreement within this range. *Insets*: actual photographs of impinged PGS2, PGS4, PGS6, PGS8, PGS10, and PGS12 materials, along with schematic representations of the photothermal heating of PGSs.

However, the effect of impinging on the PGS surface was most clearly evident with the shorter deposition time (t_deposition_ = 15 s). Comparing the top views in Figure 3a,b, a noticeable degree of aggregation is apparent due to the short post-flame annealing process. The second and most significant effect of impinging is that after this step, every PGS side-view film thickness becomes uniform and compact (see Figure 3b,d,f,h,j,l), with Ag@SiO_2_ film thicknesses varying between L_z_ = 1–23 μm. Ιn Figure S7, we plot the film thickness of impinged PGSs versus FSP deposition time, revealing a linear relationship that can be described by the linear Equation (5) as follows:

(5) $$L_{z}=0.064\cdot t_{\mathrm{deposition}}$$

with no saturation observed within this specific t_deposition_ range. Equation (5) indicates a consistent increase in film thickness with deposition time, suggesting that when combined with the impinging step, the present FSP process allows full control of the film thickness through control of t_deposition_. The thermal profiles of the films (Figure 4) indicate that the details of film-engineering affect the thermoplasmonic responses, as we analyse in full detail hereafter.

**Figure 5:**
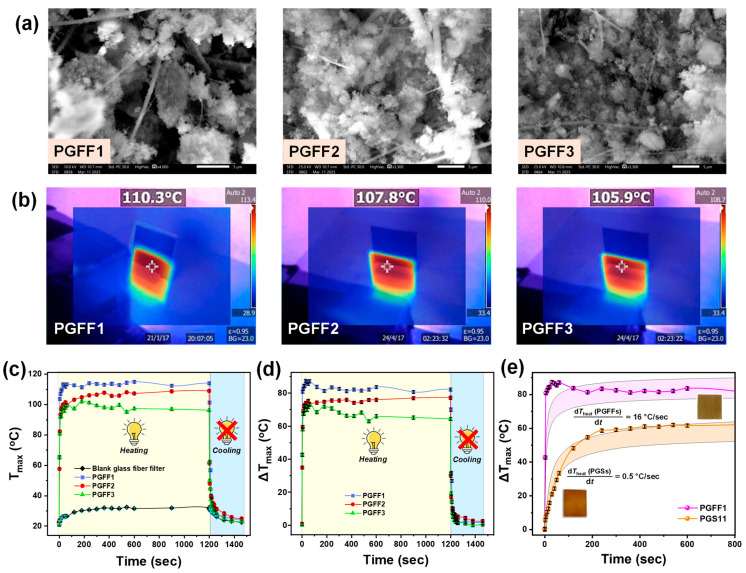
(**a**) SEM images of Ag@SiO_2_ PGFFs. (**b**) Thermal images of all PGFF materials, showing the localized maximum temperature (T_max_). (**c**) Kinetics plot of maximum temperature (T_max_) versus time for both heating (light ON from 0 to 1200 s) and cooling (light OFF from 1200 to 1400 s) processes for all PGFFs. (**d**) Time–temperature profile (normalized to the blank glass fiber filter) for all PGFF materials. (**e**) Comparison of time–temperature profiles for PGS11 and PGFF1 materials during the first 80 s of illumination. The data indicate that substrate type significantly influences photothermal efficiency, with PGFFs exhibiting heating rates 32 times faster than PGSs (PGFF1: dT/dt = 16 °C/s; PGS11: dT/dt = 0.5 °C/s).

In Figure 5a, SEM images of the PGFF materials are presented. The images reveal that Ag@SiO_2_ nanoparticles are lodged between the glass fibers. Following the impinging process, an increase in the aggregation of nanoparticles was observed. However, compared with the PGSs, the surface of the PGFFs appears to have a more isolated distribution of nanoparticles. This can be attributed to the structural differences between the two substrates. PGFFs consist of an intertwined glass fiber network, creating a porous, three-dimensional-net with multiple anchoring points, leading to a more dispersed and aggregation-based distribution. In contrast, the PGS materials provide a flat/continuous surface. This exemplifies the versatility of FSP as a tool for engineering nanofilms with different micro-morphologies. For reference, SEM images of the blank glass substrates and blank glass fiber filters are shown in Figures S5 and S6 in the Supporting Information.

### 3.2. Thermoplasmonic Performance of Ag@SiO_2_ PGSs and PGFFs: Impact of Impinging and Substrate Type on Heat Generation Dynamics

In this subsection, we investigate the thermoplasmonic performance of plasmonic glass substrates (PGSs) and plasmonic glass fiber filters (PGFFs). Specifically, we analyze the temperature increase (ΔT_max_) in both materials upon irradiation with a visible-light LED at 405 nm, exploiting the localized surface plasmon resonance (LSPR) mechanism for heat dissipation. Additionally, we assess the impact of impinging on these materials and elucidate how the substrate type (PGS or PGFF) influences photothermal generation. All thermal experiments were conducted in three independent replicates for each film to ensure reproducibility and statistically valid estimation.

#### 3.2.1. Irradiation of Ag@SiO_2_ PGSs with 405 nm LED Light

All PGS films were tested under visible-light irradiation (405 nm LED), and the temperature increase was measured *in situ* using thermal imaging camera. Under the given conditions, we measured the power density of the 405 nm LED source to be 0.14 ± 0.01 W/cm^2^ (comparable to the AM1.5 solar irradiance at the Earth’s surface) [73], and this was maintained constant throughout all our thermal experiments. As shown in Figure 4a,b, the film temperatures (T_max_) were found to vary with film thickness, with T_max_ = 70 ± 1 °C for the thinner film (PGS2: 1.1 μm) and T_max_ = 99 °C for the thicker film (PGS12: 23 μm), respectively. As a background measurement, the blank glass substrate (25 × 30 × 2 mm) yielded a ΔΤ_max_ = 13 ± 1 °C. Therefore, in Figure 4c, we present the time−temperature evolution profile of all the PGSs; the thinnest films exhibited a net heat generation (relative to a reference) of ΔT_max_ = 35 ± 1 °C (Figure 4c), whereas increasing the thickness of the Ag@SiO_2_ film allowed ΔT_max_ to reach up to 60 ± 1 °C. The thickest films functioned as the most efficient heaters, as they contained a higher density of Ag@SiO_2_ nanoparticles on the same surface area (see SEM results in Figure 3), leading to enhanced thermal interactions between neighboring particles. In our case, the dense arrangement of Ag nanoparticles within the film, facilitated strong plasmonic coupling and promoted multi-charge accumulation upon light excitation. This local charge build-up enhanced non-radiative plasmon decay via electron–phonon interactions, leading to more efficient conversion of absorbed light into heat. Consequently, the observed increase in ΔT during illumination can be attributed to this mechanism. This phenomenon aligns with prior studies [74], where closely packed plasmonic structures demonstrated improved photothermal performance due to enhanced charge storage and energy dissipation pathways.

The effect of the impinged film thickness (L_z_) on the thermoplasmonic performance is presented in Figure 4d. To clarify, we focused only on the impinged films, as the SEM results (Figure 3) showed that they were homogeneous and compact. To simulate the curve behavior, we used parameterization to obtain a nonlinear curve fit in exponential form. Then, we merged the theoretical steady-state collective contribution term for 2D finite-size square film on a substrate, given by Equation (2), with the derived exponential term. Consequently, the empirical expression that characterizes the ΔT profile as a function of Ag@SiO_2_ film thickness (L_z_) is formulated as follows:

(6) $${\Delta T}_{\mathrm{heating}}{\;(p,\;L}_{z})=\frac{\sigma_{\mathrm{abs}\;}I}{\kappa_{\mathrm{eff}}}\;\frac{\ln(1+\sqrt{2})}{\pi }\;\frac{S}{p^{2}}\;\left\{C\cdot \exp\left[-\frac{3}{{2\;(L}_{z}+1)}\right]\right\}$$

where *C* is the constant derived from parameterization and is equal to 0.077 ± 0.003. In Figure 4d, the green-colored belt refers to Equation (6); within this belt, the theoretical and experimental data show agreement. The width of the theoretical belt arises from the use of a representative range of values for interparticle distance and film thickness. In our calculations, the interparticle distance (p) was fixed at 2 nm, based on previous work [50]. Through this analysis (Figure 4d), we observed a trend featuring [i] a monotonic increase in ΔT_max_ with L_z_, up to L_z_ = 30 μm, while [ii] for L_z_ > 30 μm, ΔT_max_ appeared to reach a plateau. The observed limit at L_z_ = 30 μm corresponds to the optimal optical opacity introduced by the thicker films.

The cooling process at light-off is depicted by the bluish regions in Figure 4b,c. An increase in the thickness of Ag@SiO_2_ films led to slower cooling rate, attributed to the enhanced thermal insulation properties of thicker films, which impeded efficient heat dissipation. It is important to note that, according to the theoretical background [13], both the heating and cooling processes can be successfully described and simulated using Equations (3) and (4).

#### 3.2.2. Irradiation of Ag@SiO_2_ PGFFs with 405 nm LED Light

In Figure 5b, we display thermal images of the PGFFs, showing the temperatures (T_max_) ranging from 106 to 110 °C. As reference, the blank glass fiber filter (25 × 30 mm) exhibited ΔΤ_max_ = 8 ± 1 °C. In Figure 5c, we illustrate the T_max_ kinetics over time, displaying both heating and cooling curves, while in Figure 5d, the time−temperature evolution profile of the PGFFs is presented; the non-impinged PGFF1 material exhibited a net heat generation of ΔT_max_ = 87 ± 1 °C, which was 27 °C higher than that of the best PGS material (PGS11).

The present data reveal an intriguing pattern; PGFF materials exhibit higher photothermal efficiency than PGSs, implying the previously underappreciated role of substrate type in the microscopic monitoring of thermoplasmonic processes. Additionally, analysis of the time-temperature profiles for both PGS and PGFF materials (Figure 4b,c and Figure 5c,d) revealed that all the materials experienced a slight decrease in thermoplasmonic performance, after impinging step. This observation is further discussed in the subsequent subsection.

#### 3.2.3. Effects of Impinging

If we define the following parameter:

(7) $${\Delta T}_{\mathrm{impinging}}={\Delta T}_{\max}^{\;\mathrm{no}-\mathrm{impinging}}-{\Delta T}_{\max}^{\;\mathrm{with}\;\mathrm{impinging}}$$

then, our data show ΔT_impinging_ = −3.4 ± 1 °C on average for PGSs and ΔT_impinging_ = −10 ± 1 °C for PGFFs. Hence, we conclude that the impinging process affects negatively the photothermal yield (Figure 4b,c and Figure S7). This can be attributed to flame-induced aggregation of silver nanoparticles during impinging, where controlled aggregation can enhance photothermal properties through plasmonic coupling; however, excessive aggregation leads to negative effects. Some of these effects include the following: [i] enhanced aggregation alters the interparticle spacing, disrupting the collective oscillation of conduction electrons (LSPR), which is crucial for efficient photothermal conversion; [ii] the impinging-induced aggregation of Ag nanoparticles results in a red shift in the absorbance wavelength, moving the LSPR peak away from the optimal wavelength for photothermal absorption, thereby reducing photothermal efficiency; [iii] enhanced aggregation results in a decrease in the surface area-to-volume ratio of the nanoparticles, limiting the number of active sites available for light interaction. This decrease in active surface area further diminishes the photothermal conversion efficiency of the substrate.

#### 3.2.4. Effect of Substrate Type

Our data reveal that the structural differences between substrates play a crucial role in determining their photothermal response (Figure 5e). Specifically, in Figure 5e, if we take the first data points, which lie within the linear region of temperature increase, and perform linear fits, the slopes give $\frac{{\mathrm{dT}}_{\mathrm{heat}}}{\mathrm{dt}}=0.50\;\pm \;0.03\;{}^{\circ}C/s$ for PGSs and $\frac{{\mathrm{dT}}_{\mathrm{heat}}}{\mathrm{dt}}=16.0\;\pm \;2.3\;{}^{\circ}C/s$ for PGFFs. The faster photothermal rates observed in the PGFFs can be attributed to several key factors. First, the porous structure of the PGFFs enhanced light scattering and extended the light–matter interaction time, leading to increased absorption of incident light by the embedded Ag@SiO_2_ nanoparticles. This higher absorption efficiency translated to improved photothermal conversion. Second, the lower thermal conductivity of the glass fiber network (0.05 W m^−1^ K^−1^) compared to the bulk glass substrates (0.98 W m^−1^ K^−1^) minimized heat dissipation, allowing more localized heating and a faster increase in temperature upon illumination. Third, the distribution of isolated nanoparticles in the PGFFs reduced plasmonic coupling effects that might otherwise have led to energy losses, thereby enhancing the overall photothermal efficiency. Lastly, the increased surface area and porosity of glass fiber filters provided more effective heat transfer pathways, further contributing to the observed rapid photothermal response. Collectively, these factors explain why the PGFFs exhibited significantly faster photothermal kinetics compared with the PGSs, making them promising candidates for applications requiring efficient light-to-heat conversion. In addition to their photothermal rates, the PGFFs were more efficient in terms of temperature increase, as previously mentioned, which is evident in Figure S8, with an increase of approximately 30 °C compared with the best PGS material.

#### 3.2.5. Theoretical Evaluation

Using the theoretical Equations (3) and (4), along with the thermal properties of the medium listed in Table S1 and fitted parameters in Table S2 of the Supporting Information, the theoretical values of ΔT as a function of irradiation time were calculated for one PGS (PGS11) and one PGFF material (PGFF1). The goodness of fit (R^2^) for Figure 5e was 0.89 for PGS11 and 0.84 for PGFF1. The calculated ΔΤ vs. time for the impinged PGSs is indicated by the magenta-colored (PGFF1) and orange-colored (PGS11) belts in Figure 5e. In our calculations, the interparticle distance (p) and absorption cross section (σ_abs_) were determined based on the particle size distribution of Ag@SiO_2_ nanoparticles estimated from a TEM image obtained under similar FSP conditions in our previous laboratory work [50]. This approach follows the methodology suggested by Baffou et al. [63]. It is important to note that the lower packing density of the fiber network (Table S1) accounts for the ten-fold larger inter-particle distance p assigned to the PGFF films compared to the PGS films. Despite the polydispersity of the flame-synthesized Ag nanoparticles, we can satisfactorily simulate experimental trends using Baffou’s expression [63] by assuming a fractal dimensionality for the non-monodispersed Ag@SiO_2_ aggregates deposited on the two-dimensional glass substrates. This analysis leads to the key conclusion that increasing the Ag@SiO_2_ film thickness enhances the thermoplasmonic yield (ΔΤ_max_), a finding corroborated by theoretical predictions.

#### 3.2.6. Determination of Photothermal Conversion Efficiency

Several studies [75,76] have demonstrated that the photothermal conversion efficiency of particles, defined as their ability to convert absorbed photons into heat, can be expressed by the following equation:

(8) $$\eta =\frac{{\mathrm{hS}(T}_{\max}-T_{\mathrm{amb}})-Q_{0}}{P(1-10^{-A_{\lambda }})}=\frac{{\mathrm{hS}\Delta T}_{\max}}{P(1-10^{-A_{\lambda }})}$$

where *h* is the heat transfer coefficient, *S* the surface area of the 2D film, T_amb_ the ambient temperature, fixed at 23 °C, T_max_ the maximum localized temperature measured by thermal camera, *Q*_0_ the energy input by the blank glass substrate, *P* the incident’s light power, *A*_λ_ the optical absorbance of the PGS film, and ΔT_max_ the maximum temperature increase after subtracting the contribution of the blank glass substrate (soda–lime glass) along with the ambient temperature.

The natural (free) convection heat transfer coefficient for air (h) typically ranges from 1 to 25 W m^−2^ K^−1^, depending on the specific conditions and configuration. In this work, we adopted an approximate average value of h = 15 W m^−2^ K^−1^, based on Fundamentals of Heat and Mass Transfer by Bergman and Lavine [77]. The surface area of the 2D film was 25 × 30 mm and the incident irradiation power as measured was P = 0.14 W/cm^2^. We obtained the ΔT_max_ values from the thermal experiments (see Figure 4c and Figure S8) and the A_λ_ values from the UV-Vis results (Table S3). It is important to note that photothermal conversion efficiency was calculated only for the PGS-based materials—specifically for the impinged samples—as they exhibited surface uniformity. The PGFF-based materials (i.e., glass fiber filters) were excluded due to their opacity, which prevented accurate optical absorption analysis. In Figure S9, the photothermal conversion efficiencies of the six different PGS-impinged materials are presented. The efficiencies were approximately 60%, with the highest observed in the impinged PGS2 film, which had the smallest thickness (L_z_ = 1.1 μm) and achieved an efficiency of *η* = 65%. This superior photothermal conversion efficiency observed for the thinnest film can be attributed to several factors. First, thinner films possess lower thermal mass, enabling more rapid and pronounced increases in temperature under illumination. In contrast, thicker films tend to conduct a significant portion of the absorbed heat into the underlying substrate, whereas thinner films confine heat closer to the surface, thereby enhancing the observable temperature increase. Furthermore, if the film is optically thin yet highly absorbing, most of the incident light is absorbed within a small volume, concentrating heat generation and resulting in a localized and efficient thermal response. Collectively, these factors contribute to the enhanced photothermal conversion efficiency observed in thinner films.

## 4. Conclusions

The present data reveal the efficient thermoplasmonic heat generation of Ag nanoparticles coated with an ultrathin (1–2 nm) silica (SiO_2_) nanolayer, under 405 nm LED visible light excitation. Using in situ film deposition via flame spray pyrolysis (FSP), we successfully synthesized Ag@SiO_2_ nanoplasmonic structures and deposited them onto solid substrates, including glass (PGSs) and glass fiber filters (PGFFs), forming films with precisely controlled thicknesses ranging from 1 to 23 μm, in a single-step process. The thermoplasmonic heat generation efficiency (ΔT) of these films was systematically analyzed under visible-light irradiation (λ = 405 nm). Our results indicate that film compactness of PGSs plays a critical role in heat-generation efficiency and response rate compared with PGFFs. While the impinging process enhanced thickness/homogeneity in PGSs, it decreased the photothermal yield (−3.4 °C in average for PGSs and −10 °C for PGFFs) due to increased nanoparticle aggregation, which lowered the surface area-to-volume ratio and limited active sites for light interaction. Notably, PGFFs exhibited a significantly faster rate of temperature increase, 32 times higher (dT_heat_/dt = 16 °C/s) than that of PGSs (dT_heat_/dt = 0.5 °C/s), which is attributed to the substrate type. Additionally, PGFFs achieved a higher maximum temperature (ΔT_max_), reaching 87 ± 1 °C, compared with 60 ± 1 °C for PGSs. Finally, we calculated the photothermal conversion efficiencies, which were approximately 60%, with the highest efficiency (η = 65%) observed in the thinner impinged PGS2 film. This work underscores the potential of FSP-derived Ag@SiO_2_ nanofilms as a versatile and scalable platform for thermoplasmonic heat generation applications, highlighting their industrial relevance.

## Figures and Tables

**Table 1:** FSP film deposition parameters for Ag@SiO_2_ PGSs and PGFFs.

Materials	Ring-to-Substrate/ Filter Distance (cm)	Deposition Time *t*_deposition_ (s)	Flame-Annealing/ Impinging Time *t*_impinging_ (s)	Ag Particle Diameter *d*_XRD_ (nm) (±1)	Ag@SiO_2_ Film Thickness *L*_z_ (μm)
*Plasmonic Glass Substrates (PGSs)*					
PGS1	5	15	–	20	0.8–3 (irregular)
PGS2	5	15	30	24	1.1 ± 0.3
PGS3	5	45	–	24	1.5–3.5 (irregular)
PGS4	5	45	30	25	2.7 ± 0.5
PGS5	5	60	–	22	2–5 (irregular)
PGS6	5	60	30	26	3.7 ± 0.7
PGS7	5	90	–	20	3–7 (irregular)
PGS8	5	90	30	24	5.0 ± 0.8
PGS9	5	180	–	20	6–12 (irregular)
PGS10	5	180	30	22	11.0 ± 1.2
PGS11	5	360	–	22	10–20 (irregular)
PGS12	5	360	30	24	23.0 ± 1.6
*Plasmonic Glass Fiber Filters (PGFFs)*					
PGFF1	50	–	–	20	–
PGFF2	50	–	60	20	–
PGFF3	50	–	120	21	–

## Data Availability

The original contributions presented in this study are included in the article/Supplementary Material. Further inquiries can be directed to the corresponding author.

## Supplementary Materials

The following supporting information can be downloaded at https://www.mdpi.com/article/10.3390/nano15100743/s1, Figure S1: EDS spectrum of a representative PGS material, showing the presence of both Ag and Si.; Figure S2: Thermal imaging of an infrared thermal imager (Fluke TiS40), displaying a localized temperature (T_max_) of (a) 35.7 °C for the blank glass substrate and (b) 31.1 °C for the blank glass fiber filter. The thermal gradient from red (higher temperature) to blue (lower temperature) highlights heat dissipation patterns.; Figure S3: Newport 1918-c handheld optical power meter. (b) Phoseon Technology Inc. FireJet FJ100 72 × 20 AC LED lamp, λ = 405 ± 10 nm. (c) The nanoplasmonic glass substrates (PGSs) and glass fiber filters (PGFFs) were irradiated inside an improvised enclosed box. The samples were placed on a resin base to ensure vertical alignment with the LED illumination and were fixed at the focal point of the beam. The emission window size was 75 × 20 mm. Temperature measurements were performed using a thermal imaging camera (Fluke TiS40).; Figure S4: Spectral profile of the Phoseon Technology Inc. FireJet FJ100 72 × 20 AC LED lamp with a specific wavelength of 405 ± 10 nm.; Figure S5: SEM images of the blank glass substrate: (a) top view displaying the bare surface, and (b) side view demonstrating the pristine glass layer. Inset: actual photograph of the glass substrate.; Figure S6: Top-view SEM images of the Albet LabScience GF6 glass fiber filter (257 mm in diameter): (a) showing the bare fibers; (b) magnified region of (a), illustrating pristine fibers with diameters ranging from 0.5 to 2 µm. Inset: photograph of the blank glass fiber filter.; Figure S7: Plot of Ag@SiO_2_ film thickness versus FSP deposition time. The film thickness data were obtained via SEM imaging (see main text). It is evident that there is a linear relationship between these two parameters, and no saturation was observed within this specific range. *Insets*: actual photographs of impinged PGS2, PGS4, PGS6, PGS8, PGS10, and PGS12 materials.; Figure S8: Plot of maximum temperature values (ΔT_max_) for all the Ag@SiO_2_ materials (PGSs and PGFFs). Regarding the PGSs, ΔT_max_ increased with film thickness. Additionally, a decrease in temperature was observed for each material after the impinging step.; Figure S9: Photothermal conversion efficiencies for all impinged-PGS materials, calculated via Equation (8) and the values in Table S3.; Table S1: Thermal and dielectric properties of the surrounding media at room temperature (25 °C) and atmospheric pressure (1 atm).; Table S2: Fitted parameters of calculated ΔT by Equation (2); Table S3: ΔΤ_max_ and A_λ_ values were obtained from thermal experiments and UV-Vis measurements, respectively. Refs. [56,77,78,79,80,81,82,83,84,85,86,87] are cited in the Supplementary Materials.
