# Supplementary material for: Thermoplasmonic Nano–Hybrid Core@Shell Ag@SiO2 Films Engineered via One–Step Flame Spray Pyrolysis
Source: Nanomaterials (Basel). 2025 May 15;15(10):743. doi: 10.3390/nano15100743 (PMC12114082; doi:10.3390/nano15100743)
Supplement: Supplementary file 1 [file nanomaterials-15-00743-s001.zip › nanomaterials-3606939-supplementary.pdf]

# Supporting Information

# Thermoplasmonic Nano–Hybrid Core@Shell $Ag@SiO_2$ Films Engineered via One–Step Flame Spray Pyrolysis

**Christos Dimitriou and Yiannis Deligiannakis ***

Laboratory of Physical Chemistry of Materials & Environment, Department of Physics, University of Ioannina, 45110 Ioannina, Greece; ch.dimitriou@uoi.gr
* Correspondence: ideligia@uoi.gr; Tel.: +30-2651008662

# Supplementary Figures

## Supplementary Figure S1

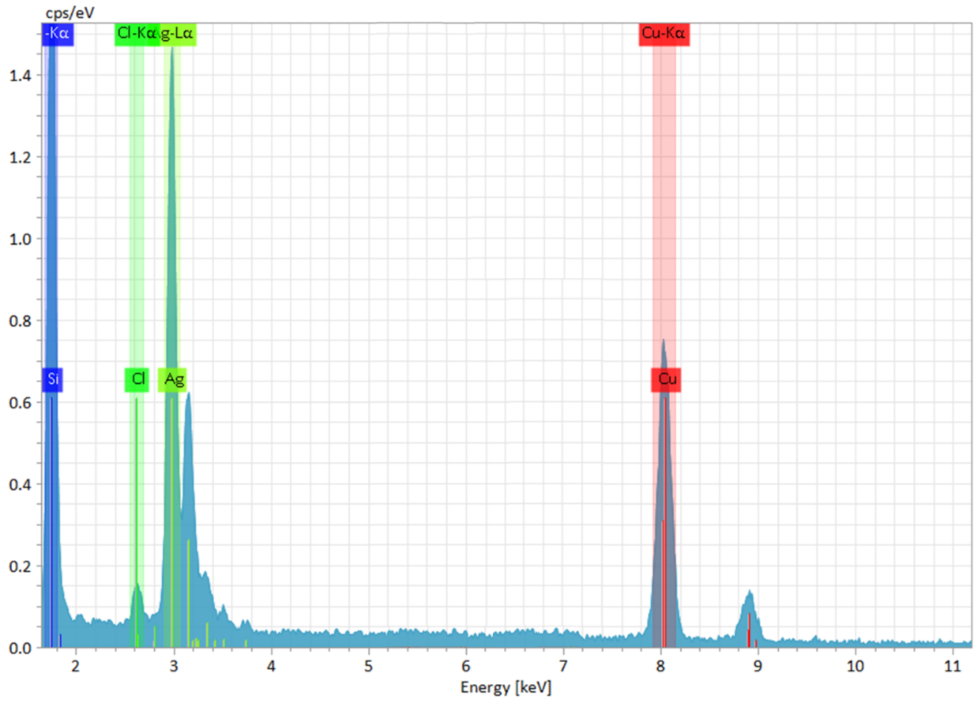


**Figure S1.** EDS spectrum of a representative PGS material, showing the presence of both Ag and Si.

## Supplementary Figure S2

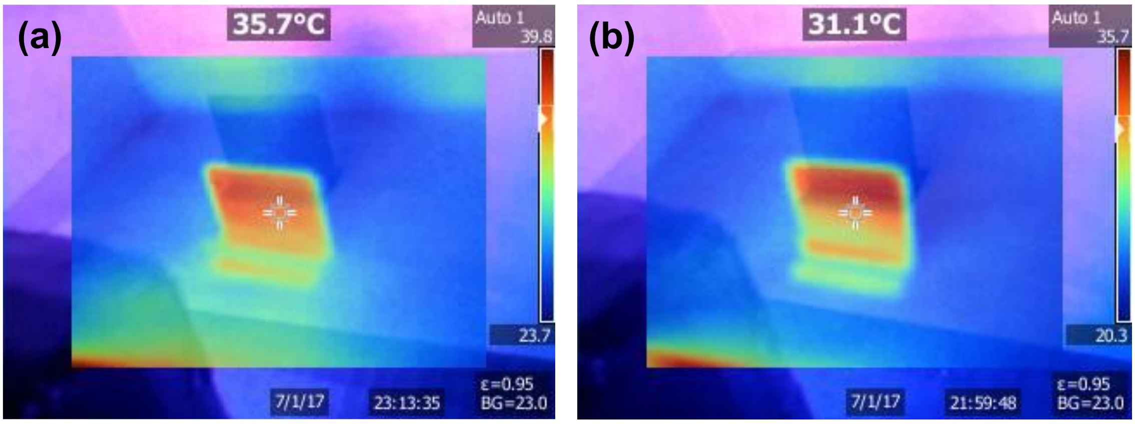


**Figure S2.** Thermal imaging of an infrared thermal imager (Fluke TiS40), displaying a localized temperature ($T_{max}$) of (a) 35.7 °C for the blank glass substrate and (b) 31.1 °C for the blank glass fiber filter. The thermal gradient from red (higher temperature) to blue (lower temperature) highlights heat dissipation patterns.

## Supplementary Figure S3

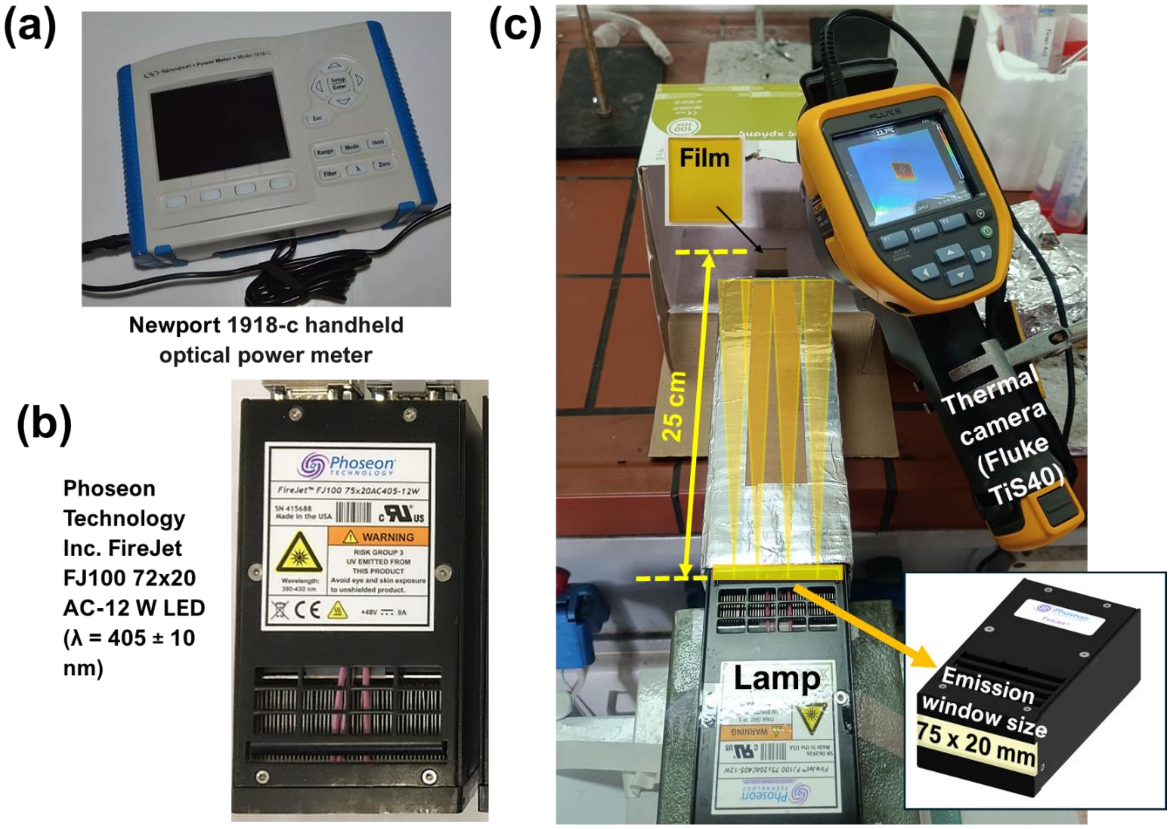


**Figure S3.** (a) Newport 1918-c handheld optical power meter. (b) Phoseon Technology Inc. FireJet FJ100 72×20 AC LED lamp, λ = 405 ± 10 nm. (c) The nanoplasmonic glass substrates (PGSs) and glass fiber filters (PGFFs) were irradiated inside an improvised enclosed box. The samples were placed on a resin base to ensure vertical alignment with the LED illumination and were fixed at the focal point of the beam. The emission window size was 75 × 20 mm. Temperature measurements were performed using a thermal imaging camera (Fluke TiS40).

## Supplementary Figure S4

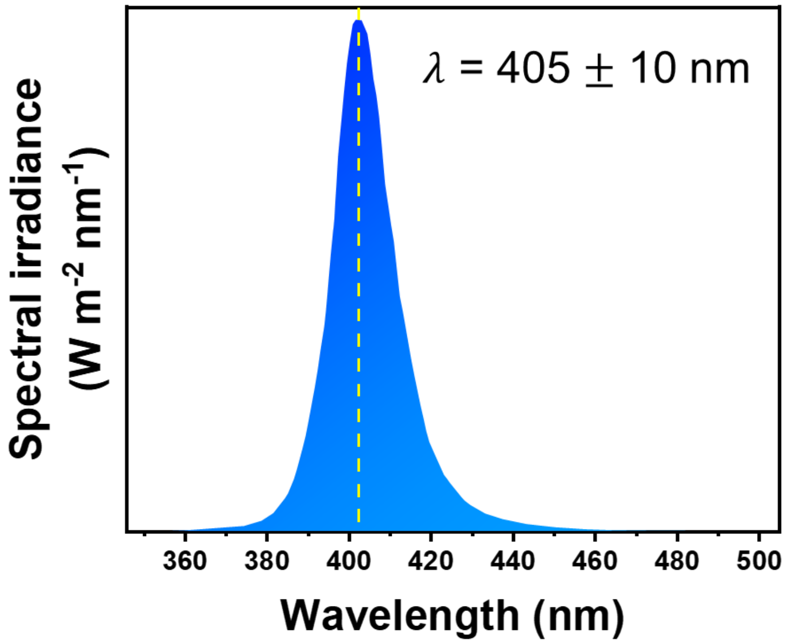


**Figure S4.** Spectral profile of the Phoseon Technology Inc. FireJet FJ100 72×20 AC LED lamp with a specific wavelength of 405 ± 10 nm.

## Supplementary Figure S5

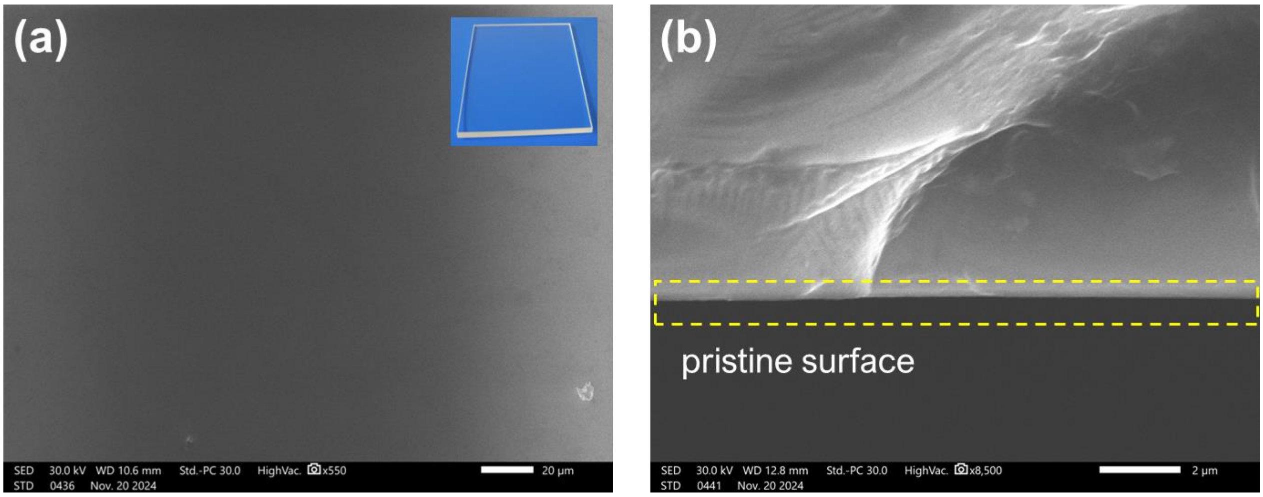


**Figure S5.** SEM images of the blank glass substrate: (a) top view displaying the bare surface, and (b) side view demonstrating the pristine glass layer. *Inset*: actual photograph of the glass substrate.

## Supplementary Figure S6

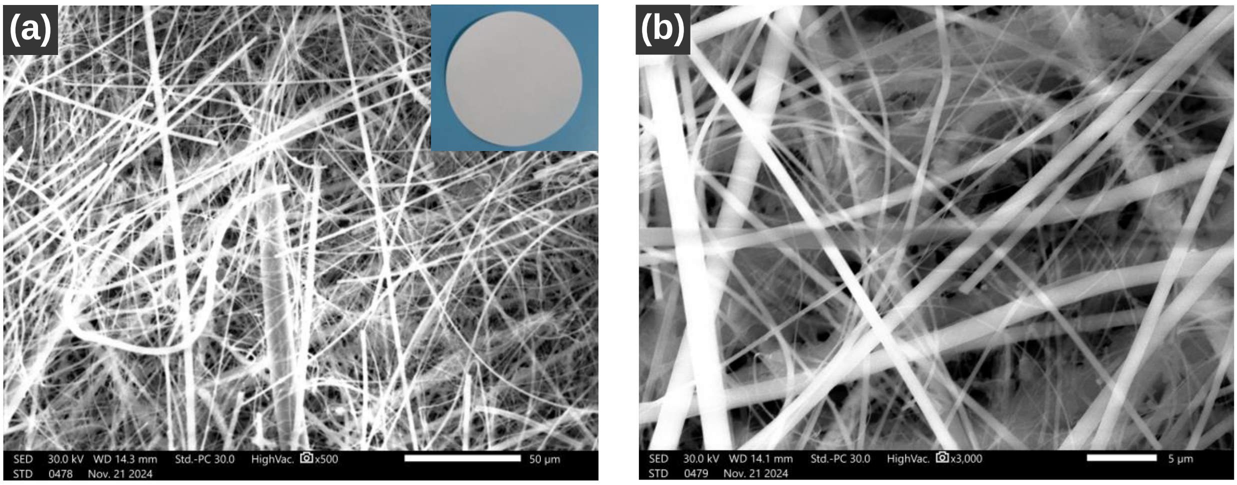


**Figure S6.** Top-view SEM images of the Albet LabScience GF6 glass fiber filter (257 mm in diameter) (a) showing the bare fibers; (b) magnified region of (a), illustrating pristine fibers with diameters ranging from 0.5 to 2 µm. *Inset*: photograph of the blank glass fiber filter.

## Supplementary Figure S7

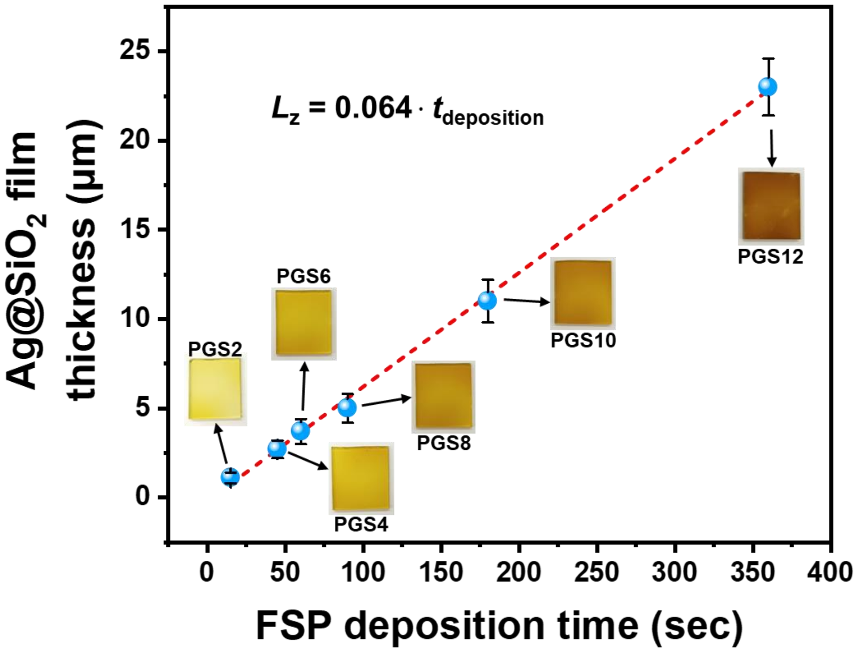


**Figure S7.** Plot of $Ag@SiO_2$ film thickness versus FSP deposition time. The film thickness data were obtained via SEM imaging (see main text). It is evident that there is a linear relationship between these two parameters, and no saturation was observed within this specific range. *Insets*: actual photographs of impinged PGS2, PGS4, PGS6, PGS8, PGS10, and PGS12 materials.

**Supplementary Figure S8**

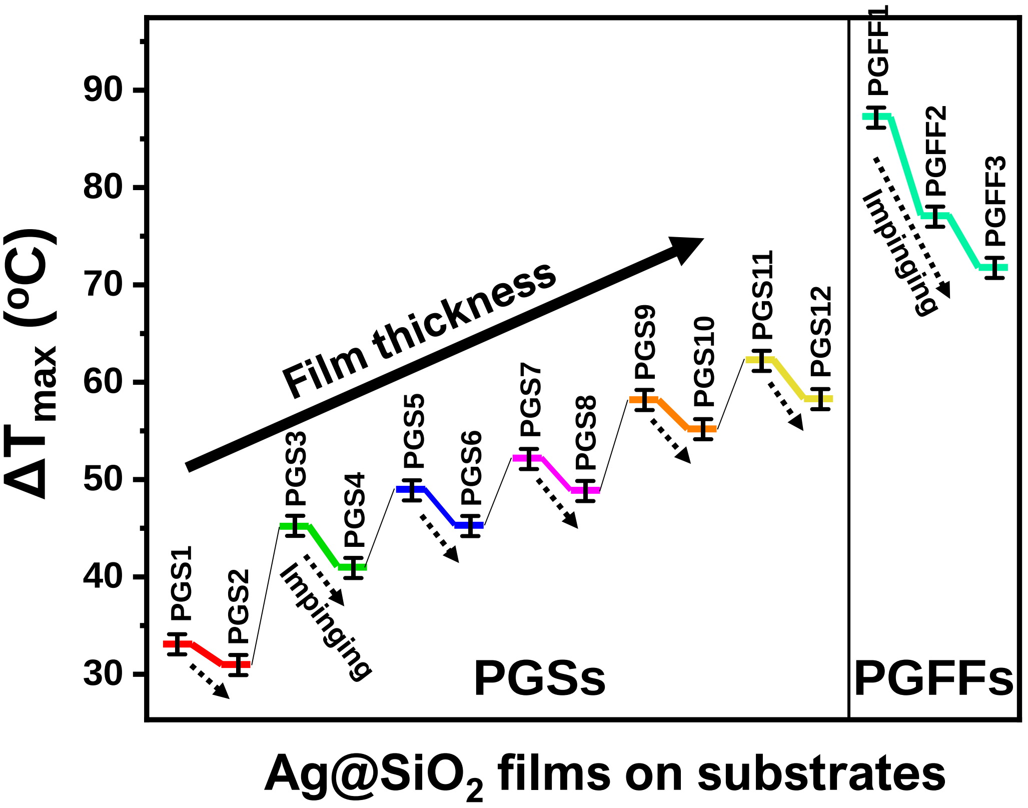


**Figure S8.** Plot of maximum temperature values ($\Delta T_{max}$) for all the Ag@$SiO_2$ materials (PGSs and PGFFs). Regarding the PGSs, $\Delta T_{max}$ increased with film thickness. Additionally, a decrease in temperature was observed for each material after the impinging step.

## Supplementary Figure S9

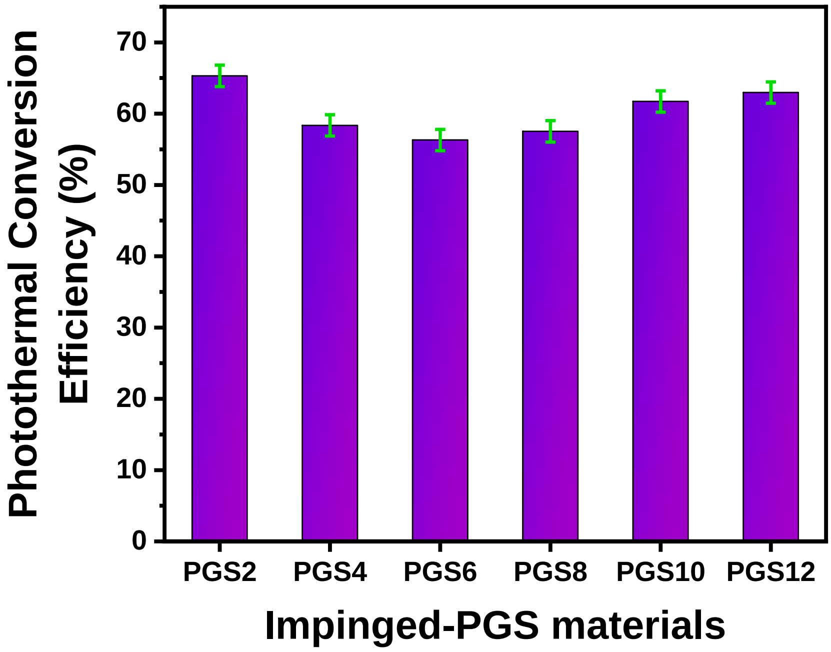


**Figure S9.** Photothermal conversion efficiencies for all impinged-PGS materials, calculated via Eq.(8) and the values in Table S3.

# Supplementary Tables

## Supplementary Table S1

**Table S1.** Thermal and dielectric properties of the surrounding media at room temperature (25 °C) and atmospheric pressure (1 atm).

| **Medium** | **Thermal conductivity ($W\ m^{-1}\ K^{-1}$)** | **Specific heat capacity ($J\ kg^{-1}\ K^{-1}$)** | **Density ($kg\ m^{-3}$)** | **Dielectric constant $\varepsilon_r$** |
|---|---|---|---|---|
| Air | 0.0263–0.0264[1,2] | 1007[1] | 1.225–1.293[3,4] | 1.0006[2] |
| Soda–lime glass | 0.937–1[5,6] | 840[6] | 2500–2550[6,7] | 7.2[8] |
| Glass fiber | 0.05[9] | 830[10] | 229[*] | 5.0[11] |

# Supplementary Table S2

**Table S2.** Fitted parameters of calculated ΔT by Eq. 2.

| Material | Absorption cross-section $\sigma_{abs}$ ($nm^2$) | Interparticle distance p (nm) | Distance from the heat source r (mm) |
|---|---|---|---|
| PGS11 | 6649 ± 650 | 2 ± 0.5 | 7 ± 0.5 |
| PGFF1 | 6080 ± 600 | 20 ± 1 | 0.3 ± 0.1 |

The fitting was performed using Origin 2018 software, with the error function selected as the objective function. The convergence criteria were set as follows: parameter tolerance = $10^{-8}$, $\chi^2$ tolerance = $10^{-8}$, derivative tolerance = $10^{-8}$, and a maximum of 400 iterations.

**Table S3.** $\Delta T_{max}$ and $A_\lambda$ values were obtained from thermal experiments and UV-Vis measurements, respectively.

| Material | $\Delta T_{max}$ (°C) | $A_\lambda$ (arb. un.) |
|---|---|---|
| PGS2 | 58.3 ± 1 | 2.10 ± 0.11 |
| PGS4 | 55.2 ± 1 | 1.38 ± 0.16 |
| PGS6 | 48.9 ± 1 | 1.05 ± 0.08 |
| PGS8 | 45.3 ± 1 | 0.86 ± 0.10 |
| PGS10 | 41.4 ± 1 | 0.62 ± 0.13 |
| PGS12 | 31.1 ± 1 | 0.31 ± 0.07 |

Since some values in Table S1, specifically those for the glass fiber filter (Hahnemühle FineArt, Inc.) were not publicly available, we refer to values for similar materials, such as borosilicate glass.

*For the density of glass fiber filter, we know that the Hahnemühle GF 6 glass fiber filter possesses the following physical properties[12]:

- Basis weight: 80 g/m$^2$
- Thickness: 0.35 mm

To estimate the density of the GF 6 filter, we used the provided basic weight and thickness:

$$Density = \frac{Basis\ weight}{Thickness} = \frac{80\ \mathrm{g\ m^{-2}}}{0.00035\ \mathrm{m}} = 228.6\ \mathrm{kg\ m^{-3}}$$

This calculated density of approximately 229 kg/m$^3$ reflects the porous structure of the glass fiber filter, which is significantly less dense than solid glass (~2500 kg/m$^3$).

## MATLAB code for calculating the absorption cross-section ($\sigma_{abs}$) of a silver (Ag) nanosphere

The following MATLAB code simulates a metallic Ag nanosphere under irradiation by an incoming plane wave, computing the scattering cross-section for different light wavelengths using the full Maxwell equations via a boundary element method (BEM) simulation:

```
%% initialization
% options for BEM simulation
op = bemoptions( 'sim', 'ret', 'interp', 'curv' );

% table of dielectric functions
epstab = { epsconst( 4.375 ), epstable( 'silver.dat' ) };

% diameter of sphere
diameter = 26;

% initialize sphere
p = comparticle( epstab, { trisphere( 144, diameter ) }, [ 2, 1 ], 1, op );
plot( p, 'EdgeColor', 'b' );

%% BEM simulation
% set up BEM solver
bem = bemsolver( p, op );

% plane wave excitation
exc = planewave( [ 1, 0, 0; 1, 0, 0 ], [ 0, 0, 1; 0, 0, 1 ], op );
% light wavelength in vacuum
enei = linspace( 300, 700, 80 );
% allocate scattering and extinction cross sections
sca = zeros( length( enei ), 2 );
ext = zeros( length( enei ), 2 );

multiWaitbar( 'BEM solver', 0, 'Color', 'g', 'CanCancel', 'on' );
% loop over wavelengths
for ien = 1 : length( enei )
% surface charge
sig = bem \ exc( p, enei( ien ) );
% scattering and extinction cross sections
abs( ien, : ) = exc.abs( sig );
ext( ien, : ) = exc.ext( sig );

multiWaitbar( 'BEM solver', ien / numel( enei ) );
end
% close waitbar
multiWaitbar( 'CloseAll' );
%% final plot
figure(2);
plot( enei, abs, 'o-' ); hold on;
```

```
xlabel( 'Wavelength (nm)' );
ylabel( ' cross section (nm^2)' );
```

As shown in the above MATLAB code, the absorption cross-section depends on the dielectric environment and the diameter of the plasmonic particle (highlighted in bold red). The dielectric constant was estimated as the average of either the air–glass or air–glass fiber system (see Table S1).

## References


(1) Bergman, T. L.; Lavine, A. S. *Fundamentals of Heat and Mass Transfer*, 8th Edition.; Wiley: United States of America, 2017.

(2) Haynes, W. M. *CRC Handbook of Chemistry and Physics, 97th Edition*; CRC Press, 2017.

(3) Ulazia, A.; Sáenz, J.; Ibarra-Berastegi, G.; González-Rojí, S. J.; Carreno-Madinabeitia, S. Global Estimations of Wind Energy Potential Considering Seasonal Air Density Changes. *Energy* **2019**, *187*, 115938. https://doi.org/10.1016/j.energy.2019.115938.

(4) Earth Science Data Systems, N. *Air Mass/Density | NASA Earthdata*. https://www.earthdata.nasa.gov/topics/atmosphere/air-mass-density (accessed 2025-05-03).

(5) Pilkington North America. *Properties of Soda-Lime-Silica Float Glass (ATS-129)*. Technical Bulletin. https://www.google.com/url?sa=t&rct=j&q=&esrc=s&source=web&cd=&ved=2ahUKEwiV17zpnomNAxX0cfEDHQCOAcYQFnoECBYQAQ&url=https%3A%2F%2Fwww.pilkington.com%2F-%2Fmedia%2Fpilkington%2Fsite-content%2Fusa%2Fwindow-manufacturers%2Ftechnical-bulletins%2Fats129propertiesofglass20130114.pdf&usg=AOvVaw1QcY7Pwa7wjo8gAsis4j3d&opi=89978449 (accessed 2025-05-04).

(6) McLellan, G. W.; Shand, E. B. *Glass Engineering Handbook*, Subsequent edition.; McGraw-Hill: New York, NY, 1984.

(7) Varshneya, V. K.; Mauro, J. C. *Fundamentals of Inorganic Glasses*, 3rd Edition.; Elsevier, 2019.

(8) Hampton Research Corp. *Soda-Lime Glass | Technical Data Sheet*. https://hamptonresearch.com/uploads/support_materials/Glass_10_Soda_lime_glass_Glass_0100_Technical_Data_Sheet.pdf (accessed 2025-05-04).

(9) Modarresifar, F.; Bingham, P. A.; Jubb, G. A. Thermal Conductivity of Refractory Glass Fibres. *J Therm Anal Calorim* **2016**, *125* (1), 35–44. https://doi.org/10.1007/s10973-016-5367-0.

(10) Continental Trade Sp. z o.o. *Borosilicate glass | Technical Data*. https://www.continentaltrade.com.pl/en/our-offer/technical-glass/types-of-materials/borosilicate-glass (accessed 2025-05-04).

(11) Tamura, S. Low-Dielectric-Constant Glass Fiber and Glass Fiber Fabric Made Thereof. US6846761B2, January 25, 2005. https://patents.google.com/patent/US6846761B2/en (accessed 2025-05-04).

(12) Hahnemühle FineArt, Inc. *Filter Papers & Membranes*. Industry & Laboratory | Product Profile & Application. https://www.hahnemuehle.com/fileadmin/user_upload/bilder/Filtration/pdf/web-10603856_Katalog_Filtration_EN.pdf (accessed 2025-03-12).
